# Enterocyte-Specific Inactivation of SIRT1 Reduces Tumor Load in the APC^+/min^ Mouse Model

**DOI:** 10.1371/journal.pone.0066283

**Published:** 2013-06-14

**Authors:** Vid Leko, Gemma J. Park, Uyen Lao, Julian A. Simon, Antonio Bedalov

**Affiliations:** 1 Clinical Research Division, Fred Hutchinson Cancer Research Center, Seattle, Washington, United States of America; 2 Human Biology Division, Fred Hutchinson Cancer Research Center, Seattle, Washington, United States of America; 3 Departments of Medicine and Biochemistry, University of Washington, Seattle, Washington, United States of America; University of Pittsburgh, United States of America

## Abstract

SIRT1 is a mammalian NAD^+^-dependent histone deacetylase implicated in metabolism, development, aging and tumorigenesis. Prior studies that examined the effect of enterocyte-specific overexpression and global deletion of SIRT1 on polyp formation in the intestines of APC^+/min^ mice, a commonly used model for intestinal tumorigenesis, yielded conflicting results, supporting either tumor-suppressive or tumor-promoting roles for SIRT1, respectively. In order to resolve the controversy emerging from these prior *in vivo* studies, in the present report we examined the effect of SIRT1 deficiency confined to the intestines, avoiding the systemic perturbations such as growth retardation seen with global SIRT1 deletion. We crossed APC^+/min^ mice with mice bearing enterocyte-specific inactivation of SIRT1 and examined polyp development in the progeny. We found that SIRT1-inactivation reduced total polyp surface (9.3 mm^2^ vs. 23.3 mm^2^, p = 0.01), average polyp size (0.24 mm^2^ vs. 0.51 mm^2^, p = 0.005) and the number of polyps >0.5 mm in diameter (14 vs. 23, p = 0.04), indicating that SIRT1 affects both the number and size of tumors. Additionally, tumors in SIRT1-deficient mice exhibited markedly increased numbers of cells undergoing apoptosis, suggesting that SIRT1 contributes to tumor growth by enabling survival of tumor cells. Our results indicate that SIRT1 acts as a tumor promoter in the APC^+/min^ mouse model of intestinal tumorigenesis.

## Introduction

SIRT1 is a mammalian NAD^+^-dependent histone deacetylase that plays important roles in ageing, metabolism, development, neurodegeneration and tumorigenesis (reviewed in [1]–[6]). Considering the multitude of cellular pathways it affects, SIRT1 appears to play a rather complex role in the biology of cancer, and evidence supports both tumor promoting and tumor suppressing functions [7], [8].

SIRT1 was first implicated in tumorigenesis by the finding that it deacetylates and down-regulates the tumor suppressor p53 under conditions of genotoxic stress, decreasing its pro-apoptotic activity and promoting survival of cells that have accumulated DNA damage [9], [10]. Acetylation of p53 turned out to be a critical posttranslational modification, one that controls many functions of the p53 protein [11], [12]. SIRT1 was later found to deacetylate and regulate several other proteins that share similar roles in cellular stress responses (e.g. Ku70, p73, FoxO3a, FoxO4 and E2F1[13]–[17]), while small-molecule inhibitors of SIRT1 were shown to exhibit antitumor activity, suggesting that pharmacological inhibition of SIRT1 could be therapeutically beneficial in a subset of human cancers [18]–[23]. SIRT1 has also been proposed to participate in tumorigenesis through epigenetic silencing of tumor suppressor genes [24]. Coupled with the observation that SIRT1 expression levels are increased in many human tumors (e.g. colon cancer) and usually associated with poor prognosis in these patients [25]–[31], these findings suggest that SIRT1 acts as a tumor promoter.

Paradoxically, a growing body of evidence suggests that SIRT1 may suppress tumor development. SIRT1 was found to be important for maintaining genome stability, loss of which is a hallmark of cancer [32], and to mediate DNA damage repair [33], [34]. Furthermore, global overexpression of SIRT1 led to reduced incidence of some age-related tumors and protection from metabolic-syndrome driven liver cancer [35]. SIRT1^+/−^ p53^+/−^ mice developed spontaneous tumors at higher rates than their p53^+/−^ controls [33], while p53^+/−^ mice overexpressing SIRT1 demonstrated decreased incidence of thymic lymphoma and increased survival following γ-radiation [32]. Despite the results of these animal studies, mutations in SIRT1 gene have never been documented in human tumors, indicating that SIRT1 may not behave as a typical tumor suppressor. However, consistent with its potential anti-oncogenic role, SIRT1 expression was found to be decreased in a subset of human cancers [33], [36].

The APC^+/min^ mouse model mimics the early events of colon cancer in humans [37] and is widely used to test the effects of potential oncogenes and tumor suppressors on formation of intestinal tumors. Heterozygous APC^+/min^ mice inherit a nonsense mutation in one copy of the tumor suppressor gene APC (designated as APC^min^) and lose the remaining wild-type allele after birth, resulting in nuclear translocation and constitutive activation of the crucial Wnt-signaling effector β-catenin and subsequent formation of numerous tumors (polyps) in the intestines [38], [39]. When a SIRT1 transgene was overexpressed in the intestinal epithelium, APC^+/min^ mice developed fewer intestinal polyps, which was attributed to the increased deacetylation and nuclear exclusion of β-catenin [40] and subsequently decreased proliferation rates of the tumor cells. However, since the expression of exogenous SIRT1 in the enterocytes surpassed physiological levels by several fold and because gene overexpression can sometimes phenocopy gene loss-of-function through a dominant interfering effect, it was important to examine the effect of SIRT1 deficiency on polyp formation in the same model. Intriguingly, APC^+/min^ mice exhibiting whole-body SIRT1 deficiency developed a similar number of intestinal polyps as the APC^+/min^ controls, but demonstrated significantly decreased average polyp size, indicating that SIRT1 could actually promote tumor growth [41]. However, as it was known that SIRT1-null mice that survived weaning exhibit severe growth retardation and decreased circulatory levels of free Insulin-like Growth Factor 1 (IGF-1), a known promoter of tumor growth [33], [42]–[44], concerns were raised that systemic adaptations to global SIRT1 deficiency may have masked the true effect of intestinal SIRT1-deficiency and protected these animals from tumor development.

In order to eliminate potentially confounding influences of global SIRT1 deletion and resolve the controversy regarding the exact role of SIRT1 in intestinal tumorigenesis, we crossed APC^+/min^ mice with mice harboring SIRT1-inactivation restricted to the intestinal epithelium. Unlike their whole-body deficient counterparts, mice with intestine-specific SIRT1 deletion demonstrated wild-type expression of SIRT1 in extra-intestinal tissues and exhibited normal survival rates without detectable developmental defects. We found that these mice exhibited significant reduction in polyp sizes and developed significantly fewer polyps >0.5 mm in diameter than their controls. Additionally, tumors from SIRT1-deficient mice exhibited significantly increased apoptotic rates, without affecting their proliferation, indicating that SIRT1 promotes survival of tumor cells. Taken together, our findings indicate that SIRT1 plays a tumor-promoting role in the mouse intestinal tumor model.

## Results

**Figure 1:**
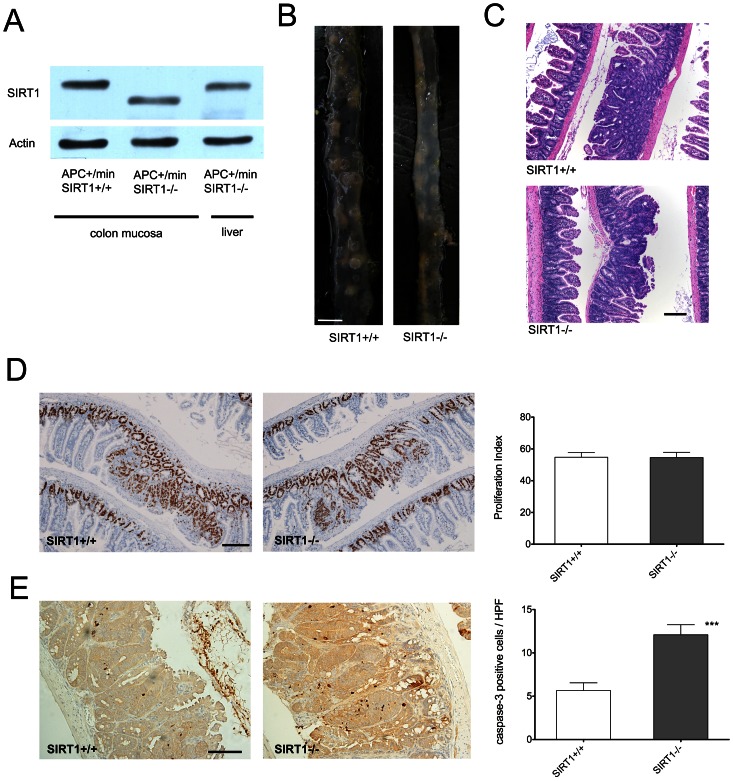
Enterocyte-specific SIRT1 deletion increases the rate of apoptosis in the intestinal tumors of APC^+/min^ mice. (A) A representative western blot showing expression of the wild-type SIRT1 protein in the intestinal epithelium of APC^+/min^ SIRT1^+/+^ mice (first lane) and a truncated version in the epithelium of APC^+/min^ SIRT1^−/−^ mice (second lane). Liver cells of the APC^+/min^ SIRT1^−/−^ mice (third lane), as well as other tissues (not shown) express the wild-type protein. Pan-actin immunostaining served as a loading control. (B) Representative photographs of unfixed small intestines (distal segments) showing similar polyp number for APC^+/min^ SIRT1^+/+^ (right) and APC^+/min^ SIRT1^−/−^ (left) mice. Scale bar indicates 5 mm length. (C) Representative photomicrographs of typical polyps from the two groups of mice, stained with hematoxylin and eosin. Scale bar indicates 100 µm length. (D) Representative photomicrographs and a bar graph showing Ki-67 immunohistochemical staining of polyp sections from APC^+/min^ SIRT1^+/+^ (left) and APC^+/min^ SIRT1^−/−^ (middle) mice. Scale bar indicates 100 µm length. Proliferation index for polyps from APC^+/min^ SIRT1^+/+^ and APC^+/min^ SIRT1^−/−^ mice (right), expressed as a fraction of Ki-67 positive cells within each polyp. Bars represent means ± SEM, n = 20 polyps per group. No statistically significant difference was observed. (E) Representative photomicrographs and a bar graph showing activated (cleaved) caspase-3 immunohistochemical staining of polyp sections from APC^+/min^ SIRT1^+/+^ (left) and APC^+/min^ SIRT1^−/−^ (middle) mice. Scale bar indicates 100 µm length. Absolute numbers of apoptotic (caspase-3 positive) cells per high power field (400 x) for polyps from SIRT1^+/+^ and SIRT1^−/−^ mice (right). Bars represent means ± SEM, n = 25 polyps per group. ***p<0.001.

We generated mice in which endogenous SIRT1 has been specifically inactivated in the intestinal epithelium using the Villin-Cre system [45] and crossed them with APC^+/min^ mice to generate APC^+/min^ SIRT1^−/−^ (APC^+/min^ Sirt^co/co^ Vil-Cre^+/−^, or SIRT1^−/−^) and APC^+/min^ Sirt1^+/+^ (APC^+/min^ Sirt^co/co^ Vil-Cre^−/−^, or SIRT1^+/+^) mice. APC and Cre genotypes were confirmed by PCR prior to weaning, while the deletion of SIRT1 was confirmed at the time of sacrifice by a western blot analysis of intestinal mucosa scrapings. A truncated version of the SIRT1 protein confirmed that exon 4 of SIRT1 gene, which encodes the catalytic domain of the protein, was successfully deleted in the enterocytes. Other tissues from APC^+/min^ SIRT1^−/−^ mice exhibited a band of the wild-type size, confirming that the deletion was intestine-specific (Figure 1A). Importantly, we noted that APC^+/min^ SIRT1^−/−^ mice were born at expected Mendelian frequencies and did not exhibit increased postnatal mortality or developmental defects and phenotypic features of mice with systemic deletion of SIRT1.

**Figure 2:**
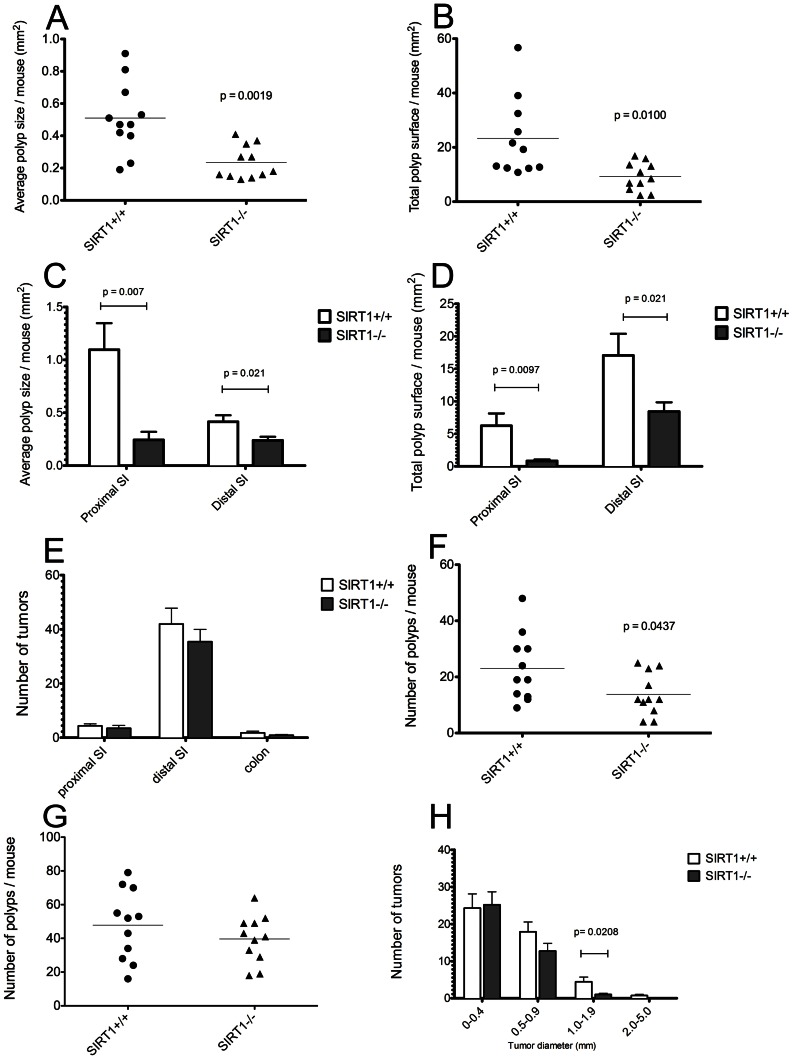
Enterocyte-specific SIRT1 deletion reduces tumor size and number of tumors >0.5 mm in diameter. (A) Average surface area of polyps in the small intestine. Total polyps surface area for each mouse was divided by total number of polyps to obtain average polyp surface area. Each dot represents an individual animal; n = 11 per group, p = 0.00019. (B) Total surface area of polyps in the small intestine; samples are as in panel A. Polyp surface was calculated as a square of tumor radius multiplied by π; surface areas of all the polyps in each mouse were then added up to obtain total polyp surface. Each dot represents an individual animal; n = 11 per group, p = 0.01. (C) Average surface area of polyps according to the location in the small intestine. Bars represent means ± SEMs, n = 11 per group. (D) Total surface area of polyps according to the location in the small intestine. Bars represent means ± SEMs. (E) Distribution of tumors according to specific intestinal location. No statistically significant differences were observed. (F) Number of polyps >0.5 mm in diameter for the entire small intestine; the groups are the same as in panel A. Each dot represents an individual animal; n = 11 per group, p = 0.044. (G) Total number of the polyps in the intestines of APC^+/min^ SIRT1^−/−^ (gray) and APC^+/min^ SIRT1^+/+^ mice (white). Each dot represents an individual animal; n = 11 per group. No statistically significant difference was observed. (H) Number of tumors in the whole small intestine according to diameter. Bars represent means ± SEMs; n = 11 per group. Significant difference in tumor number in APC^+/min^ SIRT1^−/−^ (gray) and APC^+/min^ SIRT1^+/+^ was observed only for 1.0–1.9 mm polyp size.

At the age of 120 days, SIRT1^−/−^ and SIRT1^+/+^ mice were sacrificed and their entire intestines examined for polyps (Fig. 1B,C). We found that the average size of the largest polyp in SIRT1^−/−^ cohort was significantly smaller than in the control group (1.3±0.6 mm vs. 2.2±0.9 mm, p<0.05) and that polyps from SIRT1^−/−^ mice exhibited a marked decrease in average (0.235 vs. 0.510 mm^2^, Figure 2A) and total surface areas (9.3 vs. 23.3 mm^2^, Figure 2B) throughout the small intestine (Figure 2C, D). The majority of tumors were found to reside in the distal segment of the small intestine, while only a few were detected in the colon (Figure 2E). Of note, tumors in the colon were excluded from the analysis because their peduncular appearance made the assessment of the exact tumor size difficult (although SIRT1^−/−^ mice had qualitatively smaller polyps in the colon as well). When we compared tumor size distribution between SIRT1^−/−^ and SIRT1^+/+^ mice we found that only 2% of adenomas in SIRT1^−/−^ mice were larger than 1.0 mm in diameter, whereas 12% of tumors in the control group reached this size (p = 0.0076). We found that the number of polyps in the small intestine that surpassed 0.5 mm was also significantly lower in SIRT1^−/−^ animals (Figure 2F), while the total polyp number and the number of very small tumors, measuring 0.4 mm or less in diameter, was not significantly different between two genotype groups (Figure 2G, H). These results indicate that SIRT1 deficiency reduces overall polyp burden in the intestine of APC^+/min^ mice primarily by curtailing their size.

We next examined expression of proliferative and apoptotic markers within size-matched tumors from SIRT1^−/−^ and SIRT1^+/+^ animals. According to staining for the proliferation marker Ki-67, we observed no differences in the proliferation rates between the two genotype groups (Figure 1D). However, polyps from SIRT1^−/−^ animals exhibited an increased number of cells expressing the apoptotic marker cleaved caspase-3 (Figure 1E), indicating that the observed reduction in tumor size is attributable to increased apoptosis of SIRT1^−/−^ cells, which is consistent with previously published findings in colon cancer cell lines [15].

**Figure 3:**
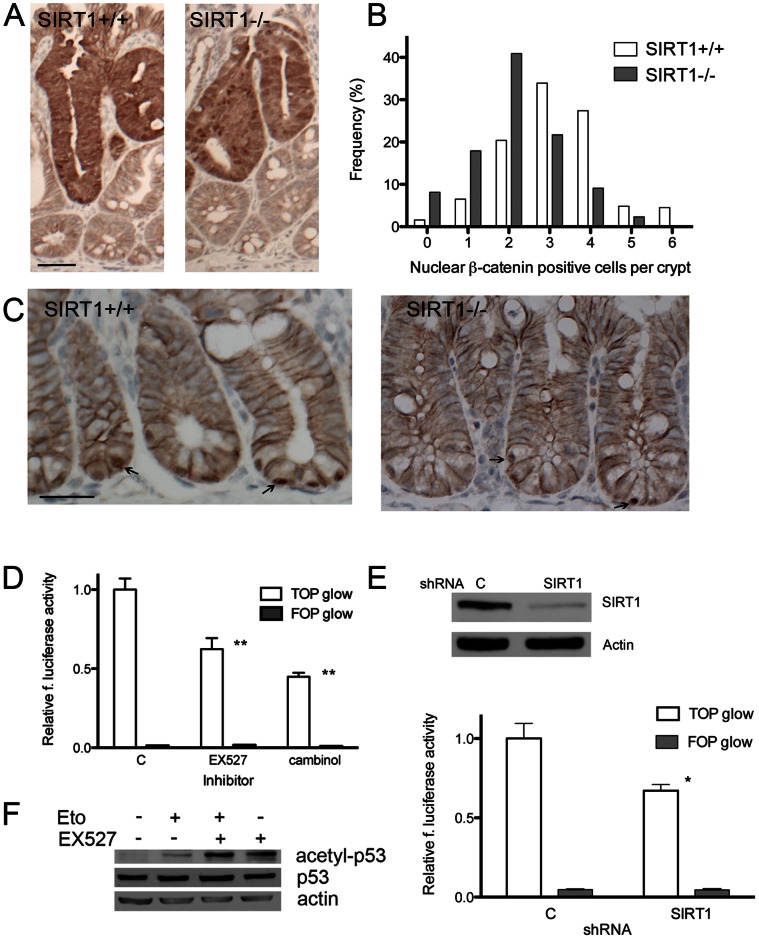
SIRT1 inactivation inhibits Wnt and promotes p53 a pathway. **A)** Representative photomicrographs showing β-catenin immunohistochemical staining of intestinal sections from APC^+/min^ SIRT1^+/+^ and APC^+/min^ SIRT1^−/−^ mice. Scale bar indicates 50 µm length. Polyps from both groups demonstrate intense cytoplasmic and nuclear staining for β-catenin. B) Bar graph with frequencies of crypts with indicated number of cells with nuclear β-catenin staining in normal appearing mucosa of APC^+/min^ SIRT1^+/+^ and APC^+/min^ SIRT1^−/−^ mice. The average number of β-catenin positive cells per crypt is reduced in APC^+/min^ SIRT1^−/−^ mice compared to APC^+/min^ SIRT1^+/+^ animals 2.1±1.2 vs 3.2±1.3 (p = 1.5×10^−5^, at least 100 crypts per genotype were scored). C) Representative photomicrographs of normal appearing mucosa from APC^+/min^ SIRT1^+/+^ and APC^+/min^ SIRT1^−/−^ mice demonstrating a reduced number of basal cells with nuclear β-catenin in APC^+/min^ SIRT1^−/−^ animals. Arrows are directed toward representative basal cells with nuclear β-catenin. Scale bar indicates 25 µm length. D) Inhibition of SIRT1 with EX527 (2 µM) and cambinol (50 µM) reduces activity of the TCF/LEF driven firefly luciferase reporter (TOP FLASH) transiently transfected into SW480 cells. TOP FLASH luciferase reporter contains minimal promoter along with three TCF binding sites, which have been mutated in FOP FLASH reporters. Bars represent means ± SEM of relative firefly luciferase activity normalized to renilla luciferase activity from the thymidine kinase promoter-driven renilla reporter that was co-transfected with TOP FLASH and FOP FLASH reporters. Each transfection is carried out in quadruplicate. **p<0.01. E) SW480 cells with shRNA-mediated downregulation of SIRT1 (top: western blot for SIRT1 and actin) exhibit reduced activity of the transiently transfected TCF/LEF driven firefly luciferase reporter (TOP FLASH). Bars represent means ± SEM of relative firefly luciferase activity normalized to renilla luciferase activity from the thymidine kinase promoter-driven renilla reporter that was co-transfected with TOP FLASH and FOP FLASH reporters. Each transfection is carried out in quadruplicate. *p<0.05. F) Immunoblot for acetyl-p53, p53 and actin from cells treated with etoposide, SIRT1 inhibitor EX527 or the combination of the two drugs. Inhibition of SIRT1 leads to p53 hyperacetylation in Hct116 colon cancer cell line. Hyperacetylation of p53 is observed in cells treated with EX527 and a combination of EX527 and etoposide. Etoposide alone modestly induces p53 acetylation.

In order to gain an insight into a mechanism by which SIRT1 promotes intestinal tumorigenesis, we analyzed the effect of SIRT1 inhibition on Wnt and p53 signaling pathways. We first analyzed β-catenin expression in adenomas of APC^+/min^ mice and observed increased expression levels and nuclear accumulation of β-catenin regardless of the intestinal SIRT1 deletion (Figure 3A). Next we wanted to determine whether loss of SIRT1 affected Wnt signaling in normal appearing intestinal crypts, where the remaining wild-type copy of the APC gene keeps overall nuclear β-catenin levels low and confined to the cell membrane in all the non-basal cells. We compared the number of nuclear β-catenin -positive cells per crypt in mice with and without intestinal SIRT1 deletion, and found that mice with the deletion had significantly fewer cells per crypt then their wild-type counterparts (Figure 3B,C), suggesting the SIRT1serves as an activator of Wnt signaling. Furthermore, to measure the effect of SIRT1 inhibition on β-catenin mediated transactivation, we analyzed the transcriptional output of T-cell factor/lymphoid enhancer factor (TCF/LEF)-driven luciferase reporter gene using a TOP-GLOW assay [46] in SW480 colon cancer cell line, which is known to exhibit a loss-of-function mutation in the APC gene and constitutive activation of Wnt pathway [47]. SIRT1 inhibition with both EX527, a selective small-molecule SIRT1 inhibitor [48], and cambinol, a non-selective SIRT1 and SIRT2 inhibitor [18], led to reduction in TCF-mediated transcription (Figure 3D). We also observed decreased TCF/LEF transcriptional activity in SW480 cells upon shRNA-mediated downregulation of SIRT1 protein level (Figure 3E), further indicating that SIRT1 activates Wnt signaling and consecutively promotes intestinal tumorigenesis in the APC^+/min^ mouse model.

Next we assessed the role of p53 acetylation in SIRT1-driven intestinal tumorigenesis by employing Hct116 colon cancer cell line with constitutive activation of β-catenin but without mutations in p53 gene [49]. As previously shown in other cell culture models [18], we saw that inhibition of SIRT1 in these colon cancer cells lead to p53 hyperacetylation (Figure 3F), suggesting that downregulation of p53 could be an additional cellular mechanism by which SIRT1 promotes tumor growth.

Taken together, our results show that enterocyte-specific inactivation of SIRT1 reduces tumor load in the intestines of APC^+/min^ mice by decreasing both overall tumor size and the number of larger tumors, and they suggest that SIRT1 acts as a tumor promoter by suppressing apoptosis of tumor cells in this mouse model, through mechanisms that include both activation of Wnt signaling pathway and inhibition of p53 activity.

## Discussion

In order to bypass systemic effects related to global deletion of SIRT1, especially those associated with reduced circulatory levels of IGF-1, and focus instead on its intestine-specific role only, we generated APC^+/min^ mice harboring enterocyte-specific inactivation of SIRT1 and examined their susceptibility to forming intestinal polyps. These APC^+/min^ SIRT1^−/−^ mice, which were born at expected Mendelian frequencies and demonstrated unperturbed development and growth, exhibited a significant decrease in the number of tumors >0.5 mm in diameter, a dramatic reduction in average and total polyp surface areas, and an increased fraction of apoptotic cells within polyps. We also found that SIRT1^−/−^ mice had similar frequencies of very small tumors as SIRT1^+/+^ controls and similar total numbers of polyps, indicating that SIRT1 could play a role in tumor progression and growth, rather than influencing the formation of early neoplastic lesions. This phenotype is congruent with one previously reported for systemic SIRT1 knockout animals [41], which showed that tumor size is significantly decreased upon SIRT1 inactivation, whereas the total number of intestinal polyps remained the same. This surprisingly robust finding shows that SIRT1 inactivation, regardless of differences in genetic background (C57BL/6 with some contribution of 129X1/SvJ vs. mixed C57BL/6/129X1/SvJ/CD1) and age of sacrifice (4 months vs. 12 months) in these two studies, leads to marked changes in tumor growth rate. Our results suggest that the tumor attenuation previously observed in APC^+/min^ mice carrying a systemic deletion of SIRT1 [41] was a tissue-specific effect of SIRT1-deficiency. However, these findings are seemingly at odds with the findings of Firestein et al. [40], which showed that intestine-specific overexpression of SIRT1 reduced the number of polyps in the intestines of APC^+/min^ mice (tumor size was not analyzed). Thus, SIRT1 overexpression and SIRT1 loss-of-function resulted in similar phenotypes, a phenomenon frequently observed in gene overexpression studies (reviewed in [50]) that may be explained by changes in the stoichiometry of protein-protein interactions and disassembly of multi-protein complexes leading, seemingly paradoxically, to inhibition of the overexpressed protein. The facts that SIRT1 functions in several multi-protein complexes and that its expression in the study by Firestein et al. [40] surpassed physiological levels by several fold make this explanation highly plausible. However, we noted that intestine-specific SIRT1 overexpression in the study by Firestein et al. did not create an exact phenocopy of the enterocyte-specific deletion, as the total number of polyps was reduced 3–4 fold in the former study, while our model demonstrated only 2 fold reduction among tumors >0.5 mm in size, without affecting the total number of tumors. Furthermore, unlike in the SIRT1-overexpression study, which did not analyze cell survival and apoptosis, we found no differences in proliferation rates between SIRT1^−/−^ and SIRT1^+/+^ mice, but rather an increased rate of apoptosis in the SIRT1^−/−^ group. This is consistent with the current paradigm in which SIRT1 plays a role as a pro-survival and anti-apoptotic mediator, as discussed in the next section. It is possible that supra-physiological levels of SIRT1 in the overexpression study engaged cellular mechanisms outside the classical SIRT1-mediated signaling pathways that are normally not active when SIRT1 is expressed at physiological levels. Based on cell culture studies, Firestein et al. attributed the polyp number reduction to reduction in transcriptional activity of β-catenin, which is a target of Wnt signaling. In contrast, our results suggest that SIRT1 actually promotes Wnt signaling, both *in vivo* and *in vitro*. Apart from the use of different cell lines, we cannot account for the discrepancy between the two *in vitro* results. However, as indicated by a growing body of evidence, the role of SIRT1 in Wnt signaling appears to be complex, as different studies had also associated SIRT1 with activation of the Wnt pathway, as discussed in greater detail below. As for the discrepancy in the *in vivo* results, we do not believe that the observed differences in proliferation rates between the two studies are attributable to the age of the animals or differences in the intestinal sections scored, as in both studies polyp analysis was carried out throughout the small intestine of the animals sacrificed at the same age. However, there is a possibility that the disparity in proliferation rates could be at least partially due to subtle differences in genetic backgrounds, as the Firestein study was carried out in C57BL/6 mice, while our study utilized C57BL/6 animals with a contribution from the 129X1/SvJ background.

Several previously described activities of SIRT1, particularly those related to modulation of p53 activity [9], [10], c-myc [51], [52], [53], [54], canonical Wnt signaling [55] and epigenetic control [24], may account for the reduction in tumor size we observed in the intestines of APC^+/min^ SIRT1^−/−^ mice. SIRT1 has been shown to promote cell survival during genotoxic stress through deacetylation and down-regulation of p53 and several other stress-response proteins (reviewed in [4]). Moreover, SIRT1 was also implicated in promoting intestinal tumorigenesis through its interactions with the tumor suppressor HIC1 (Hypermethylated in Cancer 1), a transcriptional repressor frequently inactivated in human colon cancer though epigenetic means, namely CpG island hypermethylation of its promoter [56], [57]. Heterozygosity for HIC1 in mice with a mutation in APC was shown to induce transcriptional de-repression of SIRT1 in enterocytes and to promote polyp formation, possibly through down-regulation of p53. Intriguingly, both HIC1 and SIRT1 have been shown to be direct transcriptional targets of p53, suggesting the presence of a HIC1-SIRT1-p53 autoregulatory loop. Our observation that intestinal SIRT1-deficiency decreases polyp load and increases apoptosis in the APC^+/min^ model, and that pharmacological inhibition of SIRT1 in APC-mutated colon cancer cell lines leads to p53 hyeperacetylation, provide additional support for importance of such an autoregulatory loop in the intestinal tumorigenesis.

SIRT1 was previously proposed to directly deacetylate β-catenin, which, depending on cell context, may promote or inhibit β-catenin activity [40], [58]. Besides participating in β-catenin deacetylation, there are several indirect mechanisms by which SIRT1 may promote Wnt signaling. SIRT1 downregulates the activity of p53, which has been shown to downregulate Wnt signaling by promoting β-catenin phosphorylation and degradation [59]. HIC1, whose expression could be influenced by the above described autoregulatory loop, has been shown to antagonize canonical Wnt signaling by associating with TCF4 and β-catenin and sequestering them in an inactive complex within the nucleus [60]. Additionally, SIRT1 has been shown to up-regulate all three mammalian Disheveled proteins and thereby promote Wnt signaling [55]. Finally, down-regulation of SIRT1 in breast and colon cancer cell lines leads to re-expression of the Wnt pathway inhibitors, SFRP1 and SFRP2 (Secreted Frizzled-like Proteins 1 and 2), which are frequently inactivated in human cancers through epigenetic means [24], as discussed in greater detail below. Our findings that the lack of SIRT1 decreases the number of basal crypt cells expressing the active form of β-catenin and that SIRT1-inhibition reduces β-catenin mediated transcriptional activity *in vitro*, suggest that SIRT1 may indeed promote adenoma formation through increased Wnt signaling. However, further work is required to determine which of the proposed pathways is critical for promoting Wnt signaling in the course of adenoma formation.

Clonal evolution, which dictates the progression from normal epithelium to polyp and ultimately invasive cancer, requires progressive accumulation of both new genetic lesions and epigenetic alterations that cause transcriptional silencing of tumor suppressor genes (reviewed in [61]). Besides serving as an established model for intestinal tumorigenesis, APC^+/min^ mice were the first animal model used to demonstrate that genetic or pharmacological interference with DNA methylation inhibits tumor formation, thus establishing a role of epigenetics in tumor formation and the feasibility of targeting epigenetic mechanisms for cancer therapy [62], [63]. Besides DNA methyl transferases and class I and II histone deacetlyases (HDAC), SIRT1 (a class III HDAC) has been shown to participate in gene silencing in colon cancer cell lines [24], both synergistically with DNA methyl transferases and independently, without appreciable alterations in DNA methylation level. Among genes repressed in a SIRT1-dependent manner was a set of genes frequently inactivated by DNA methylation in human cancers, providing direct evidence that SIRT1 controls expression of tumor suppressor genes. Besides the Wnt inhibitors SFRP1 and SFRP2, genes up-regulated upon inhibition of SIRT1 included E-cadherin, a cell adhesion mediating gene, and MLH1, a mismatch repair gene whose inactivation leads to a mutator phenotype. Together, these findings suggest that abrogation of epigenetic repression that accompanies polyp progression could be one of the mechanisms for the reduced tumor burden that we observed in APC^+/min^ SIRT1^−/−^ animals, raising the exciting possibility that SIRT1 inhibitors, similarly to inhibitors of DNA methyltransferase and class I/II HDACs, could be used for epigenetic therapy of cancer.

## Materials and Methods

### APC+/min SIRT1−/− (APC+/min Sirt1co/co Vil-cre+/−) Mice

Homozygous B6;129-*Sirt1^tm1Ygu^*/J mice carrying conditional deletion of Sirt1 exon 4 (Sirt1^co/co^, available from Jackson Laboratories, stock No. 008041), which encodes the catalytically active domain of the protein, were crossed with homozygous B6.SJL-Tg(Vil-cre)997Gum/J mice (a gift from Dr. William Grady (FHCRC), but also available from Jackson Laboratories, stock No. 004586) to generate heterozygous Sirt1^+/co^ Vil-cre ^+/−^ mice. Resulting heterozygotes were interbred to generate Sirt1^co/co^ Vil-cre ^+/−^ mice, which were subsequently crossed with SIRT 1^co/co^ mice to generate Sirt1^co/co^ Vil-cre^+/−^ mice and their littermate controls (Sirt1^co/co^ Vil-cre^−/−^). Both groups were then crossed with APC^+/min^ mice (available from Jackson Laboratories, stock No. 002020) to generate APC^+/min^ Sirt^co/co^ Vil-cre^+/−^ (APC^+/min^ SIRT1^−/−^) and APC^+/min^ Sirt1^co/co^ Vil-cre^−/−^ (APC^+/min^ SIRT1^+/+^) mice. APC and Vil-cre genotypes were confirmed by PCR carried out on DNA isolated from clippings prior to weaning (PCR primer sequences are available upon request).

### Ethics Statement

All animal work was carried out in strict accordance with the recommendations in the Guide for the Care and Use of Laboratory Animals of the National Institutes of Health. The protocol for this study was approved by the Fred Hutchinson Cancer Research Center Institutional Animal Care and Use Committee (file 1505).

### Animal Pathology and Histopathology

The gastrointestinal tract of the sacrificed 16-week old animals was promptly excised and cut, with mucosal side up, into 3 segments (colon, distal and proximal small intestine), flushed with ice cold phosphate-buffered saline (PBS, Invitrogen), pinned open and fixed in 10% formalin, neutral buffered (Sigma) for 45 minutes in a dissection tray. Fixed intestine was then examined under the dissection microscope by two researchers who, blinded from the genotyping data, counted the polyps, determined their diameter with a caliper and calculated their surface. For histopathology, fixed intestine segments were placed onto a dry board and gently rolled into Swiss rolls and placed in 10% NBF for 24 hours. The rolls were then switched over into 70% ethanol and submitted for paraffin-embedding and hematoxylin/eosin, Ki-67 (rat anti-mouse Ki67, Dako, M7249, 1∶50 dilution) and cleaved caspase-3 staining (rabbit anti-mouse, Biocare Medical, CP 229B, 1∶50 dilution). Microscopic images were obtained using Nikon E800 microscope and analyzed by two researchers independently. Fractions of Ki-67 and caspase-3 positive cells were determined using Image J program (Wayne Rasband, National Institutes of Health).

### Tissue Culture Studies

Hct116 and SW480 cell lines were obtained from ATCC and grown in Dulbecco’s Modified Eagle Medium (Life Technologies) supplemented with 10% Fetal Calf Serum. TOP GLOW reporter plasmid that contains the firefly luciferase gene under control of minimal c-Fos promoter, along with three TCF binding sites, mutated in a control FOP GLOW reporter [46], were obtained from Dr. Hans Clevers. Herpes simplex virus thymidine kinase promoter driven renilla luciferase reporter, pRL-TK (Promega) was used for transfection normalization. Transient transfections were carried out using Lipofectamine (Invitrogen) and firefly and renilla luciferase activities measured using Dual Luciferase Reporter Assay System (Promega). EX527 was obtained from Sigma and cambinol was synthesized in our laboratory. shRNA specific for SIRT1 (shRNA SIRT1-3-2, target sequence GCGGGAATCCAAAGGATAATT) was introduced into SW480 cells as a lentiviral particle using pLKO.1 vector and puromycin selectable marker.

### Western Blotting

After flushing and opening the large intestine, the mucosal surface was gently scraped off using a glass slide, snap frozen in liquid nitrogen and placed at −80°C. Upon thawing, each sample was resuspended in half its volume of RIPA buffer (1% NP-40, 0.1% SDS, 50 mM Tris-HCl pH 7.4, 150 mM NaCl 0.5% Sodium Deoxycholate,1 mM EDTA), sonicated, incubated on ice for 30 minutes and spun down to remove debris. A BCA Protein assay (Pierce) was performed to determine the protein concentration; 25 µg of each sample was then mixed with the equal amount of SDS gel loading buffer (2×) containing 100 mM Tris-Cl (pH 6.8), 4% (w/v) sodium dodecyl sulfate, 0.2% (w/v) bromophenol bluem 20% (v/v) glycerol, 200 mM DTT (dithiothreitol) and loaded onto 7.5% polyacrylamide gel. The protein was then transferred to a nitrocellulose membrane and subsequently probed with the rabbit anti-mouse SIRT1 antibody (Anti-SIRT1, Millipore 07-131). In tissue culture studies the following antibodies were used: rabbit polyclonal anti-SIRT1 (Millipore, 07-131), mouse monoclonal anti-p53 (DO-1 Santa Cruz Biotechology), rabbit polyclonal anti-acetyl p53 (Cell Signaling, Acetyl-p53 (Lys379), catalogue number 2570). Anti-actin staining was performed to ensure equal loading between the samples (Pan Actin, ACTN05, NeoMarkers).

### Statistical Methods

Standard two-tailed Student’s t-test was used for comparisons between two experimental groups, with a p value of <0.05 considered as significant.
